# Prevalence and associated factors of adolescent pregnancy among sexually active adolescent girls: Evidence from the Peruvian Demographic and Family Health Survey, 2015-2019

**DOI:** 10.12688/f1000research.108837.1

**Published:** 2022-05-25

**Authors:** Brenda Caira-Chuquineyra, Daniel Fernandez-Guzman, Adria Meza-Gómez, Beatriz Milagros Luque-Mamani, Shawny Luz Medina-Carpio, Carlos S. Mamani-García, Marilia Romani-Peña, Cristian Díaz-Vélez

**Affiliations:** 1 Facultad de Medicina, Universidad Nacional de San Agustin, Arequipa, Arequipa, 04001, Peru; 2 Grupo Peruano de Investigación Epidemiológica, Unidad para la Generación y Síntesis de Evidencias en Salud, Universidad San Ignacio de Loyola, Lima, 15001, Peru; 3 Escuela Profesional de Medicina Humana, Universidad Nacional de San Antonio Abad del Cusco, Cusco, Cusco, 0800, Peru; 4 Universidad Privada Antenor Orrego, Trujillo, 13007, Peru; 5 Instituto de Evaluación de Tecnologías en Salud e Investigación, Lima, Lima, 15001, Peru

**Keywords:** Adolescents, pregnant women, pregnancy in adolescence, pregnancy and motherhood, Peru

## Abstract

**Background:** To determine the prevalence and associated factors of adolescent pregnancy in Peru, 2015-2019.

**Methods:** A population-based analytical cross-sectional study was conducted using pooled data from the Demographic and Family Health Surveys of Peru 2015-2019. A total weighted sample of 6892 adolescent girls aged 15 to 19 years with a history of sexual intercourse were included. Finally, the adjusted prevalence ratio (aPR) with 95% confidence interval (CI) were reported to determine the factors that were significantly associated with adolescent pregnancy.

**Results:** The prevalence of adolescent pregnancy in Peru was 30.1% (95%CI: 28.4–31.8%). In the multivariable analysis; being 17-19 years (aPR: 1.38; 95%CI: 1.22–1.56), having a partner (aPR: 4.08; 95%CI: 3.46–4.81) and belonging to the Quechua ethnicity group (aPR: 1.20; 95%CI: 1.09–1.32), were associated with a higher prevalence. Whereas, having an occupation (aPR: 0.81; 95%CI: 0.75–0.88), currently studying (aPR: 0.42; 95%CI: 0.36–0.49), belonging to the second (aPR: 0.91; 95%CI: 0.84–0.98), third (aPR: 0.80; 95%CI: 0.72–0.89), fourth (aPR: 0.76; 95%CI: 0.64–0.89) and fifth (aPR: 0.55; 95%CI: 0.41–0.73) wealth quintile, initiating sexual relations between 17-19 years (aPR: 0.52; 95%CI: 0.46–0.59), perceiving a future pregnancy as a problem (aPR: 0.77; 95%CI: 0.70–0.83) and knowledge of the moment in the cycle when she could become pregnant (aPR: 0.84; 95%CI: 0.76–0.93), were associated with a lower prevalence of pregnancy.

**Conclusions:** About three in 10 adolescents who initiated their sexual life presented with at least one pregnancy. Age, marital status, employment, education, wealth, ethnicity, age at first intercourse, knowledge of when in the cycle she may become pregnant, and perception of future pregnancy were associated with adolescent pregnancy. It is necessary to increase national policies on family planning and sex education among adolescents to reduce the prevalence of adolescent pregnancy in Peru.

## Introduction

The World Health Organization (WHO) defines adolescents as those between 10 and 19 years of age. ^1^ Adolescence is a period of progression towards adulthood, necessary to reach physical, sexual, mental and social maturity. ^2^ However, it is during middle and late adolescence (15-19 years) when there is a greater development of responsibility for decisions and a greater search for autonomy. ^3^ Therefore, the culmination of this period together with pregnancy can lead to various difficulties for the adolescent and the infant, ^4^ with delayed prenatal care and a greater number of obstetric and perinatal complications ^5^ being common, as well as a higher incidence of maternal mortality. ^6^

According to the WHO, 12 million women aged 15-19 years and, approximately, 1 million girls under the age of 15 give birth each year. ^7^ Latin America and the Caribbean have the second highest adolescent fertility rate in the world. Although this rate decreased from 65.6% (2010-2015) to 60.7% (2015-2020), there are still significant variations between sub regions and countries. ^8^ In Peru, the Demographic and Family Health Survey (ENDES, for its Spanish acronym) revealed that the percentage of adolescents aged 15 to 19 years who were already mothers or were pregnant for the first time did not decrease notably and was maintained during 2014 to 2019 (prevalence of 14.6% and 12.6%, respectively). ^9^ This issue of teenage pregnancy has been observed at a higher proportion in women with low educational levels, who reside in rural areas, belong to low socioeconomic strata and according to ethnicity. ^10^^–^^12^

Although in Peru, different factors associated with teenage pregnancy have also been reported in small studies or in gray literature, ^13^^,^^14^ it is not known which factors are associated with pregnancy during middle and late adolescence, which are the groups in which teenage pregnancy is most prevalent. In addition, there is no evidence in the literature on the factors associated with teenage pregnancy, taking as the baseline population those adolescents who initiated sexual relations, which could overestimate the effects found in other studies. ^10^^–^^12^ Therefore, the aim of the present study was to estimate the proportion and factors associated with pregnancy in adolescents aged 15 to 19 years who initiated their sexual life. The identification of associated factors with adolescent pregnancy could be an input for the strengthening of policies on reproductive education and prenatal care.

## Methods

### Study design

We conducted a secondary analysis of the 2015-2019 ENDES database, developed through the National Institute of Statistics and Informatics (INEI, for its Spanish acronym) of Peru. This was a national survey whose target population was private households and their members, women aged 15-49 years, children under 5 years and one person aged 15 years and older per household. The present study used an observational study design, analytical cross-sectional type. The sections on demographic and social characteristics, reproductive history, use of contraceptive methods, pregnancy and breastfeeding, nuptiality, fertility preference, spouse's background and women's work of the Women's Individual Questionnaire, were used.

### Ethical aspects

This study did not require the approval of an ethics committee because the ENDES database is in the public domain and does not allow identification of the subjects, which maintains the corresponding confidentiality. The primary data collection was carried out with the prior signed consent of the interviewees. In addition, the present research project was registered in the “ *Plataforma de Proyectos de Investigación en Salud*” (PRISA) of “ *Instituto Nacional de Salud*” (INS) in Peru with code EI00000001763.

### Population, sample and sampling

The ENDES is a survey with annual representativeness at the national, urban-rural level, by geographic domain and for the 25 departments of Peru. The sampling design of ENDES is two-stage, probabilistic by clusters and stratified at the departmental level and by urban and rural area. The primary sampling unit was made up of the selected clusters. The secondary sampling unit was made up of the selected homes. ^15^

**Figure 1.**
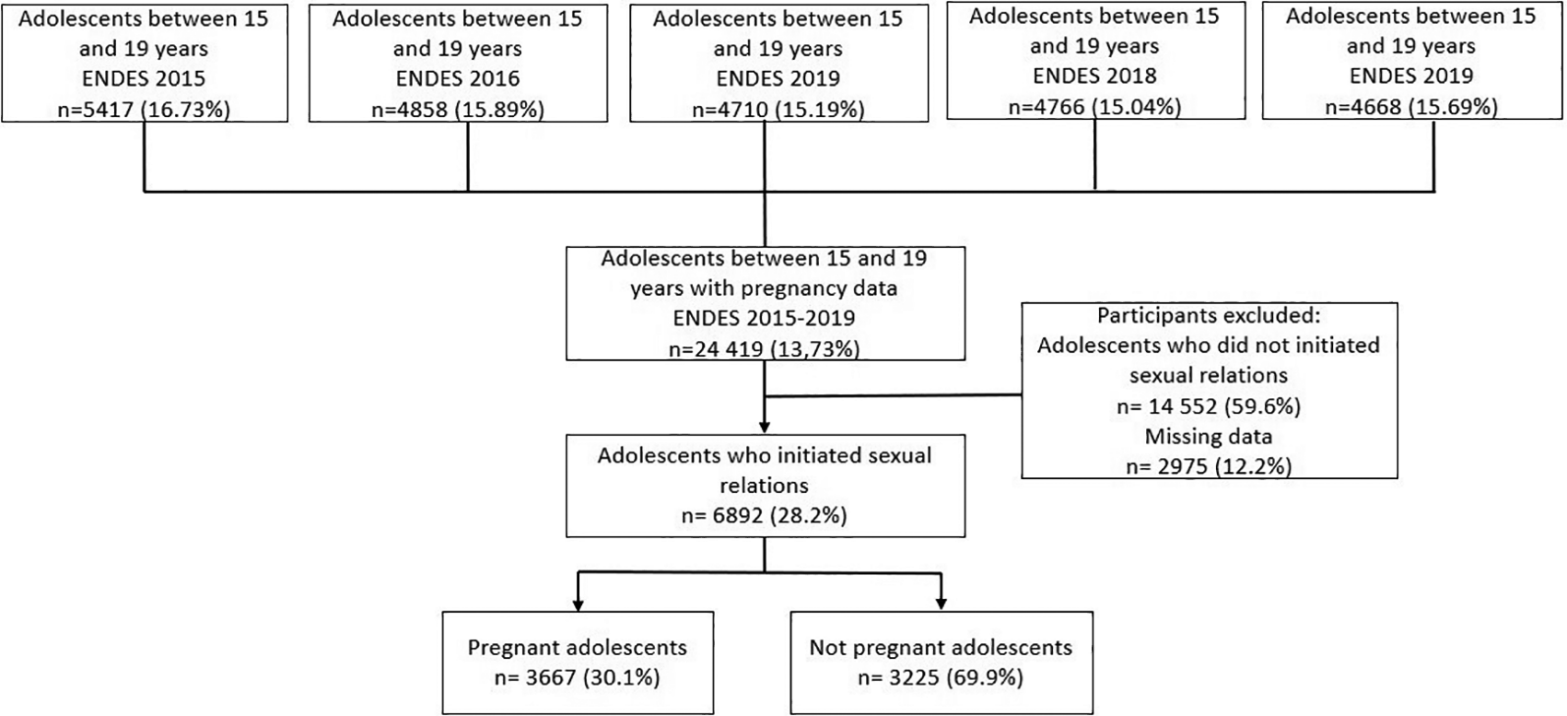
Flow chart of selection of the study sample. ENDES (Spanish acronym), Demographic and Family Health Survey.

A total of 24 419 adolescent women aged 15-19 years were surveyed during the period 2015-2019, the effective sample for the analysis was composed of 6892 women who were those who responded to the dependent variable of interest (currently pregnant or who are mothers) and who reported having initiated sexual intercourse ( Figure 1). Additional information on the ENDES survey methodology is available from Technical Report. ^15^

### Dependent variable

Adolescent pregnancy was considered as the dependent variable of the study, which was collected by self-reporting through the ENDES 2015-2019 Individual Woman Questionnaire with the following questions: “Are you currently pregnant?”, which was coded as V213 in the database (“No or not sure” / “Yes”) and the total children born, coded as V201 in the database (numerical variable), which was considered if the total number of children born was greater than or equal to 1.

### Independent variables

The following independent variables were considered: Sociodemographic variables, such as age (middle adolescence [15-16 years], late adolescence [17-19 years]), geographic region (Costa, Sierra, Selva), wealth level (first quintile, second quintile, third quintile, fourth quintile, fifth quintile), area of residence (urban, rural), ethnicity (mestizo, quechua, negro - moreno o zambo, other), presence of a partner (no, yes), education level (secondary or higher, primary or lower), current occupation (no, yes) and currently studying (no, yes), and gynecology-obstetric variables, such as age of first sexual intercourse in late adolescence (no, yes), perception of pregnancy (positive, negative), knowledge of the time of the cycle when she could become pregnant (no, yes) and use of contraceptive methods (no, yes). The selection and inclusion of these independent variables in the study was based on a review of the literature. ^10^^–^^12^

### Statistical analysis

The 2015-2019 ENDES databases were downloaded and imported into Stata ^®^ v.16.0 software (Stata Corporation, College Station, Texas, USA) (Stata, RRID:SCR_012763). All analyses were performed considering the complex sampling design for ENDES using the svy module.

For the descriptive analysis of categorical variables, absolute frequencies and weighted proportions were calculated, and for numerical variables, means with standard deviation were calculated. For bivariate analysis, the association between categorical variables was evaluated using the chi-square test. A value of p<0.05 was considered statistically significant.

To evaluate the association of interest, generalized linear models of the Poisson family with logarithmic link function were used, and we calculated crude prevalence ratios (cPR) and adjusted prevalence ratios (aPR). For the adjusted model, the method of forward manual selection and the Wald test were used to select the variables to obtain a final parsimonious model. In this way, the variables, including age, marital status, current occupation, currently studying, wealth index, ethnicity, age at first sexual intercourse, knowledge of the time of the cycle when you can get pregnant and perception of pregnancy were entered into the final model. The analyses were reported with their respective 95% confidence intervals (95% CI) and p values <0.05 were considered statistically significant. Furthermore, to examine the possible role of the area of residence as a modifier of the effect, the adjusted model was analyzed stratified into urban and rural areas.

## Results

From a total of 24 419 adolescent women aged 15 to 19 years during the study period, 14 552 were excluded because they were not at risk of becoming pregnant (no history of sexual intercourse) and 2975 were excluded because they had variables with missing data, resulting in a final study population of 6892 ( Figure 1).

The highest percentage of the study population corresponded to late adolescence (17 to 19 years) (83.2%), adolescents without a partner (67.9%), those with secondary or higher education (92.0%), those belonging to the second wealth quintile (23.1%), those currently in an occupation (58.0%), and those belonging to the mestizo ethnic group (37.8%), to the coast region (63.7%) and to an urban area (78.9%) ( Table 1).

**Table 1.** General characteristics of a subsample of Peruvian adolescent women, ENDES 2015-2019 (n=6892).

Characteristics	N	% *	95% CI *
**Age**			
15 to 16 years	1255	16.8	15.1–18.6
17 to 19 years	5637	83.2	81.4–84.9
**Marital status**			
Single	3548	67.9	65.9–69.8
Married	3344	32.1	30.2–34.1
**Educational level**			
Secondary or higher	6160	92.0	90.9–93.0
Primary or lower	732	8.0	7.0–9.1
**Current occupation**			
Does not work	3174	42.0	39.6–44.5
Works	3718	58.0	55.5–60.4
**Currently studying**			
No	4166	49.6	47.2–52.0
Yes	2726	50.4	48.0–52.8
**Region**			
Coast	2979	63.7	61.6–65.7
Saw	2226	24.4	22.7–26.1
Forest	1687	12.0	10.8–13.2
**Residence area**			
Urban	4558	78.9	77.5–80.1
Rural	2334	21.1	19.9–22.5
**Wealth index**			
First quintile	2274	20.6	19.2–22.0
Second quintile	1947	23.1	21.3–25.0
Third quintile	1274	20.9	19.0–22.9
Fourth quintile	877	19.2	17.0–21.5
Fifth quintile	520	16.3	14.1–18.7
**Ethnicity**			
Mestizo	2161	37.8	35.5–40.2
Quechua	2742	35.6	33.5–37.7
Negro/moreno/zambo	562	7.9	6.8–9.1
Other	1427	18.7	16.9–20.6
**Age first sexual intercourse**			
10 to 16 years	5221	69.2	66.9–71.3
17 to 19 years	1671	30.8	28.7–33.1
**Use of contraceptive methods**			
Do not use	2889	46.8	44.4–49.2
Used	4003	53.2	50.8–55.6
**Knowledge of the time of the cycle when you can get pregnant**			
No	5184	68.8	66.5–71.1
Yes	1708	31.2	28.9–33.5
**Perception of future pregnancy**			
A problem	5291	79.6	77.8–81.3
No problem	1601	20.4	18.7–22.2
**Teen pregnancy**			
No	3225	69.9	68.2–71.6
Yes	3667	30.1	28.4–31.8

ENDES (Spanish acronym), Demographic and Family Health Survey.

* Weighted values according to complex sampling of the survey.

The prevalence of pregnancy among adolescents aged 15 to 19 years who initiated sexual relations was 30.1%, with a higher proportion among adolescents aged between 17 to 19 years (32.0; p<0.001), those with primary education or lower (64.8%; p<0.001), those with a partner (73.3%; p<0.001), those who belonged to the first wealth quintile (54.8%; p<0.001), those who did not work (35.0%; p<0.001), those who were not studying (51.5%; p<0.001), those who belonged to the jungle region (41.5%; p<0.001), as well as those from rural areas (55.4%; p<0.001) ( Table 2).

**Table 2.** Prevalence of adolescent pregnancy according to the characteristics of the study population (n=6892).

Characteristics	Teen pregnancy						p-value
	Yes			No			
	n	%	95% CI *	n	%	95% CI *	
**Age**							
15 to 16 years	456	20.8	18.0–23.9	799	79.2	76.1–82.0	**<0.001**
17 to 19 years	3211	32.0	30.1–33.9	2426	68.0	66.1–69.9	
**Marital status**							
Without a partner	771	9.6	8.5–10.8	2777	90.4	89.2–91.5	**<0.001**
With a partner	2896	73.3	70.0–76.4	448	26.7	23.6–30.0	
**Educational level**							
Secondary or higher	3092	27.1	25.5–28.8	3068	72.9	71.2–74.5	**<0.001**
Primary or lower	575	64.8	57.8–71.3	157	35.2	28.7–42.2	
**Current occupation**							
Does not work	1828	35.0	32.3–37.8	1346	65.0	62.2–67.7	**<0.001**
Works	1839	26.6	24.5–28.7	1879	73.4	71.3–75.5	
**Currently studying**							
No	3027	51.5	48.5–54.4	1139	48.5	45.6–51.5	**<0.001**
Yes	640	9.1	8.0–10.3	2086	9.1	89.7–92.0	
**Region**							
Coast	1454	24.8	22.7–27.0	1525	75.2	73.0–77.3	**<0.001**
Highlands	1294	38.3	35.5–41.1	932	61.7	58.9–64.5	
Jungle	919	41.5	38.4–44.7	768	58.5	55.3–61.6	
**Residence area**							
Urban	2012	23.3	21.6–25.1	2456	76.7	74.9–78.4	**<0.001**
Rural	1565	55.4	52.5–58.2	769	44.6	41.8–47.5	
**Wealth index**							
First quintile	1540	54.8	51.9–57.6	734	45.3	42.4–48.1	**<0.001**
Second quintile	1089	37.9	34.4–41.5	858	62.1	58.5–65.6	
Third quintile	593	24.0	20.9–27.4	681	76.0	72.6–79.1	
Fourth quintile	318	18.0	15.1–21.3	559	82.0	78.7–84.9	
Fifth quintile	127	10.0	7.8–12.7	393	90.0	87.3–92.2	
**Ethnicity**							
Mestizo	1005	22.9	20.5–25.4	1156	77.2	74.6–79.5	**<0.001**
Quechua	1525	34.4	31.8–37.2	1217	65.6	62.8–68.2	
Negro/moreno/zambo	317	34.8	29.5–40.6	245	65.2	59.4–70.5	
Other	820	34.5	30.5–38.7	607	65.5	61.3–69.5	
**Age first sexual intercourse**							
10 to 16 years	3202	38.0	35.7–40.2	2019	62.0	59.8–64.3	**<0.001**
17 to 19 years	465	12.5	10.9–14.3	1206	87.5	85.7–89.1	
**Use of contraceptive methods**							
Do not use	1084	18.2	16.4–20.1	1805	81.8	79.9–83.6	**<0.001**
Used	2583	40.6	38.1–43.1	1420	59.4	56.9–61.9	
**Knowledge of the time of the cycle when you can get pregnant**							
No	2957	35.0	33.0–37.2	2227	65.0	62.8–67.0	**<0.001**
Yes	710	19.2	16.9–21.6	998	80.8	78.4–83.1	
**Perception of future pregnancy**							
A problem	2705	27.7	25.9–29.6	2586	72.3	70.4–74.1	**<0.001**
No problem	962	39.4	35.7–43.2	639	60.6	56.8–64.3	

* Percentages weighted according to complex survey sampling.

Furthermore, the prevalence of adolescent pregnancy was higher among those who initiated their first sexual intercourse between 10 and 16 years (38.0%; p<0.001), those who used contraceptive methods (40.6%; p<0.001), individuals who did not know the time of the cycle when they could become pregnant (35.0%; p<0.001) and those who did not perceive pregnancy as a problem (39.4%; p<0.001) ( Table 2).

When we performed the multivariate analysis, we found that the factors independently associated with a higher frequency of teenage pregnancy were as follows: Being in late adolescence (aPR: 1.38; 95%CI: 1.22–1.56), having a partner (aPR: 4.08; 95%CI: 3.46–4.81) and belong to the Quechua ethnicity group (aPR: 1.20; 95%CI: 1.09–1.32). The factors associated with a lower frequency of adolescent pregnancy were as follows: Being in an occupation (aPR: 0.81; 95%CI: 0.75–0.88), currently studying (aPR: 0.42; 95%CI: 0.36–0.49), and belonging to the second (aPR: 0.91; 95%CI: 0.84–0.98), third (aPR: 0.80; 95%CI: 0.72–0.89), fourth (aPR: 0.76; 95%CI: 0.64–0.89) and fifth (aPR: 0.55; 95%CI: 0.41–0.73) wealth quintile. Similarly, to have initiated sexual relations between 17-19 years of age (aPR: 0.52; 95%CI: 0.46–0.59), to perceive a future pregnancy as non-problematic (aPR: 0.77; 95%CI: 0.70–0.83) and to know the moment in the cycle when she could become pregnant (aPR: 0.84; 95%CI: 0.76–0.93) were also associated with a lower prevalence of teenage pregnancy ( Table 3).

**Table 3.** Factors associated with teenage pregnancy.

Characteristics	Crude model			Parsimonious adjusted model		
	cPR	95% CI	p-value	aPR	95% CI	p-value
**Age**						
15 to 16 years	Ref.			Ref.		
17 to 19 years	1.54	1.31–1.80	**<0.001**	1.38	1.22–1.56	**<0.001**
**Marital status**						
Single	Ref.			Ref.		
Married	7.62	6.66–8.71	**<0.001**	4.08	3.46–4.81	**<0.001**
**Current occupation**						
Does not work	Ref.			Ref.		
Works	0.76	0.68–0.85	**<0.001**	0.81	0.75–0.88	**<0.001**
**Currently studying**						
No	Ref.			Ref.		
Yes	0.18	0.15–0.20	**<0.001**	0.42	0.36–0.49	**<0.001**
**Wealth index**						
First quintile	Ref.			Ref.		
Second quintile	0.69	61.9–77.2	**<0.001**	0.91	0.84–0.98	**0.012**
Third quintile	0.44	37.8–50.9	**<0.001**	0.8	0.72–0.89	**<0.001**
Fourth quintile	0.33	27.4–39.5	**<0.001**	0.76	0.64–0.89	**0.001**
Fifth quintile	0.18	14.2–23.6	**<0.001**	0.55	0.41–0.73	**<0.001**
**Ethnicity**						
Mestizo	Ref.			Ref.		
Quechua	1.51	1.31–1.73	**<0.001**	1.2	1.09–1.32	**<0.001**
Negro/moreno/zambo	1.52	1.25–1.86	**<0.001**	1.04	0.91–1.19	0.536
Other	1.51	1.28–1.79	**<0.001**	1.04	0.94–1.16	0.438
**Age first sexual intercourse**						
10 to 16 years	Ref.			Ref.		
17 to 19 years	0.33	0.28–0.38	**<0.001**	0.52	0.46–0.59	**<0.001**
**Knowledge of the time of the cycle when you can get pregnant**						
No	Ref.			Ref.		
Yes	0.55	0.48–0.63	**<0.001**	0.84	0.76–0.93	**0.001**
**Perception of future pregnancy**						
A problem	Ref.			Ref.		
No problem	1.42	1.26–1.60	**<0.001**	0.77	0.70–0.83	**<0.001**

cPR: crude prevalence ratio; aPR: adjusted prevalence ratio; 95% CI: 95% confidence interval.

Prevalence ratio and confidence intervals were calculated considering complex survey sampling. The p-values <0.05 are in bold.

Factors associated with teenage pregnancy in urban and rural areas are presented in Table 4 and Table 5, respectively.

**Table 4.** Factors associated with teenage pregnancy in urban areas.

Characteristics	Crude model			Parsimonious Adjusted model		
	cPR	95% CI	p-value	aPR	95% CI	p-value
**Age**						
15 to 16 years	Ref.			Ref.		
17 to 19 years	1.64	1.31–2.06	**<0.001**	1.37	1.13–1.67	**0.001**
**Marital status**						
Single	Ref.			Ref.		
Married	9.15	7.64–10.98	**<0.001**	5.05	4.03–6.35	**<0.001**
**Current occupation**						
Does not work	Ref.			Ref.		
Works	0.76	0.65–0.88	**<0.001**	0.81	0.72–0.90	**<0.001**
**Currently studying**						
No	Ref.			Ref.		
Yes	0.17	0.14–0.21	**<0.001**	0.41	0.33–0.50	**<0.001**
**Wealth index**						
First quintile	Ref.			Ref.		
Second quintile	0.82	0.68–0.99	**0.039**	0.93	0.81–1.08	0.342
Third quintile	0.54	0.44–0.66	**<0.001**	0.84	0.72–0.98	**0.030**
Fourth quintile	0.42	0.33–0.52	**<0.001**	0.82	0.67–1.00	0.050
Fifth quintile	0.23	0.17–0.31	**<0.001**	0.62	0.45–0.85	**0.003**
**Ethnicity**						
Mestizo	Ref.			Ref.		
Quechua	1.37	1.15–1.63	**<0.001**	1.18	1.03–1.34	**0.015**
Negro/moreno/zambo	1.46	1.13–1.88	**0.004**	0.95	0.80–1.13	0.582
Other	1.23	0.96–1.57	0.095	0.99	0.84–1.17	0.913
**Age first sexual intercourse**						
10 to 16 years	Ref.			Ref.		
17 to 19 years	0.28	0.23–0.34	**<0.001**	0.48	0.40–0.58	**<0.001**
**Knowledge of the time of the cycle when you can get pregnant**						
No	Ref.			Ref.		
Yes	0.58	0.49–0.69	**<0.001**	0.84	0.73–0.97	**0.019**
**Perception of pregnancy**						
A problem	Ref.			Ref.		
No problem	1.37	1.15–1.62	**<0.001**	0.75	0.65–0.86	**<0.001**

cPR: crude prevalence ratio; aPR: adjusted prevalence ratio; 95% CI: 95% confidence interval.

Prevalence ratio and confidence intervals were calculated considering complex survey sampling. The p-values <0.05 are in bold.

**Table 5.** Factors associated with teenage pregnancy in rural areas.

Characteristics	Crude model			Parsimonious adjusted model		
	cPR	95% CI	p-value	aPR	95% CI	p-value
**Age**						
15 to 16 years	Ref.			Ref.		
17 to 19 years	1.68	1.45–1.95	**<0.001**	1.40	1.24–1.59	**<0.001**
**Marital status**						
Single	Ref.			Ref.		
Married	3.42	2.96–3.94	**<0.001**	2.38	2.05–2.76	**<0.001**
**Current occupation**						
Does not work	Ref.			Ref.		
Works	0.78	0.70–0.86	**<0.001**	0.83	0.77–0.89	**<0.001**
**Currently studying**						
No	Ref.			Ref.		
Yes	0.29	0.24–0.34	**<0.001**	0.49	0.41–0.59	**<0.001**
**Wealth index**						
First quintile	Ref.			Ref.		
Second quintile	0.85	0.74–0.97	**0.017**	0.93	0.84–1.02	0.135
Third quintile	0.85	0.61–1.19	0.346	1.02	0.82–1.26	0.869
Fourth quintile	0.37	0.13–1.01	0.052	0.88	0.47–1.65	0.685
Fifth quintile	0.12	0.01–1.07	0.058	0.35	0.07–1.84	0.214
**Ethnicity**						
Mestizo	Ref.			Ref.		
Quechua	1.20	1.04–1.38	**0.011**	1.19	1.07–1.32	**0.002**
Negro/moreno/zambo	1.17	0.94–1.46	0.150	1.16	0.97–1.38	0.094
Other	1.15	0.99–1.34	0.057	1.08	0.96–1.21	0.200
**Age first sexual intercourse**						
10 to 16 years	Ref.			Ref.		
17 to 19 years	0.55	0.47–0.64	**<0.001**	0.60	0.53–0.69	**<0.001**
**Knowledge of the time of the cycle when you can get pregnant**						
No	Ref.			Ref.		
Yes	0.69	0.59–0.80	**<0.001**	0.84	0.74–0.94	**0.003**
**Perception of pregnancy**						
A problem	Ref.			Ref.		
No problem	1.16	1.05–1.28	**0.004**	0.81	0.75–0.88	**<0.001**

cPR: crude prevalence ratio; aPR: adjusted prevalence ratio; 95% CI: 95% confidence interval.

Prevalence ratio and confidence intervals were calculated considering complex survey sampling. The p-values <0.05 are in bold.

## Discussion

In Peru, about three in 10 adolescents between 15 and 19 years of age who have initiated sexual relations have had at least one pregnancy. The high prevalence of adolescent pregnancy could favor the appearance of a higher rate of obstetric and perinatal complications in this group. ^5^ It was found in the present study that older age (17 to 19 years), the presence of a partner, ethnicity, having a job, being in school, level of wealth, early initiation of sexual relations (≤16 years), the perception of a future pregnancy as non-problematic, and knowledge of the moment in the cycle when pregnancy may occur were independently associated with adolescent pregnancy.

We found that belonging to late adolescence (between 17 to 19 years) was associated with a higher prevalence of teenage pregnancy. This is consistent with previous studies ^16^^,^^17^ and could be explained by the greater development and mental maturity in this group to assume a pregnancy, as well as a greater development of female identity and greater capacity to adapt to parenting roles. ^3^ Similarly, the literature has described a greater desire to become pregnant in late adolescence compared to the rest of adolescence. ^18^ Furthermore, it should be taken into account that the sociocultural and economic context could influence the age of onset of risky sexual behaviors in adolescents, ^19^ which could lead to a higher risk of becoming pregnant.

The presence of a partner among the adolescents was associated with a higher prevalence of teenage pregnancy. In this regard, this association was previously evidenced in studies focusing on sexually active adolescents. ^20^^,^^21^ In Latin American countries, the high prevalence of adolescent pregnancy could be due to the fact that among adolescents with a partner there is a greater desire to become pregnant and achieve motherhood in order to start a family at an early age. ^22^ In Peru, it has been reported that 69% of adolescents aged 15 to 19 years who were pregnant or had children were in some type of early union (65.8% cohabiting and 3.2% married). ^23^ Thus, the association with the presence of a partner could be due to the fact that after adolescents become pregnant, parents put pressure on the couple to marry or cohabit. Given that, family planning measures should be widely encouraged for both female and male adolescents.

We also found that belonging to the Quechua ethnicity group was associated with a higher prevalence of adolescent pregnancy. In this regard, it has been reported that women from ethnic minorities tend to experience social and economic exclusion, which could generate greater inequity in access to family planning services and contraceptive methods, ^24^^,^^25^ leading to higher maternal mortality. ^26^ In Peru in 2016, it was observed that the population of native origin (Quechua, Aymara or Amazonian origin) had a higher level of fertility and a lower proportion of contraceptive methods used. ^27^ In these ethnic groups, a greater acceptance of early marriage and pregnancy has also been reported. ^25^ Therefore, greater state intervention is required in these population groups to reduce the gaps in access to sexual and reproductive health information for adolescents.

Having an occupation or being a student was associated with a lower frequency of adolescent pregnancy. Adolescents who engage in these activities may prioritize education or economic income over starting a family. ^28^ This contrasts with previous studies conducted in middle- and low-income countries, where a higher risk of pregnancy has been reported among adolescents who do not have an occupation ^26^ or who do not attend school. ^29^ Having an education could be related to a better knowledge of sexual health and a greater ambition to complete higher education, postponing reproductive desire until greater emotional and economic stability is achieved. ^30^ In Peru, dropout at the secondary education level in 2015 was 7.6% among adolescents. ^31^ Given this, it would be important to implement national programs that promote and ensure education and find vulnerable adolescents who have dropped out of school.

It was also found in the present study that belonging to the third, fourth or fifth wealth quintile was associated with a lower frequency of adolescent pregnancy. In Latin America and the Caribbean, an early onset of sexual relations ^32^ and a higher proportion of adolescent pregnancy ^33^^–^^35^ were reported among lower income social groups. This could be explained by lower use of and limited access to contraceptive methods in these groups. ^35^ Similarly, unfavorable economic conditions could lead women to think of motherhood as a better life option, since they would have a partner to take care of household needs. ^16^^,^^17^ In Peru, the “Juntos” Program was implemented in 2005 with the aim of reducing the impact of poverty and its intergenerational transmission through the bimonthly delivery of a monetary incentive of 200 nuevos soles (S/200) to low income households, plus an additional S/100 for pregnant women who attend antenatal visit care and S/100 for each child under 3 years of age who comply with growth and development checkups. ^36^ This initiative could favor vulnerable groups such as pregnant adolescents in low socioeconomic strata.

Regarding the age of initiation of sexual intercourse, we found that late initiation of sexual intercourse (17 to 19 years) was associated with a lower prevalence of adolescent pregnancy. This finding is consistent with the literature, ^20^ which has reported earlier ages of sexual intercourse, ^32^^,^^37^ and higher proportions of a first pregnancy between 15 and 19 years of age. ^38^ This could be explained by the lack of promotion of sexual and reproductive health information, including family planning methods, at earlier ages. Therefore, the general population should be made aware of the importance of regulating access to sexual and reproductive health information from puberty and adolescence.

Another variable that was associated with a higher prevalence of adolescent pregnancy was the perception of a future pregnancy as non-problematic in the crude analysis. In this regard, it has been previously reported that a positive attitude toward pregnancy among postpartum adolescents is strongly associated with a higher prevalence of a second pregnancy. ^39^ Likewise, a greater likelihood of feeling stigmatized during pregnancy has been observed when they did not have a romantic relationship with a partner or felt verbally abused by family, friends, partners or other adolescents. ^40^ This could be due to the fact that the greater emotional stability achieved through the support of a partner and family, friends and social environment during pregnancy could generate a non-problematic perception of a subsequent pregnancy. ^41^^,^^42^ However, in our analysis, when adjusting for other variables, we found a reversal in the direction of the association. This could be explained by the fact that the sample evaluated included adolescents with no history of pregnancy, who could have a biased perception of a possible pregnancy and be influenced by the desire to start a family. This explanation is consistent with the fact that there is a high prevalence of desire for pregnancy among adolescents in Latin America. ^22^

Knowledge of the moment in the cycle when pregnancy is possible was associated with a lower prevalence of adolescent pregnancy. This is consistent with previous studies, where ignorance of the fertile days was a risk factor for adolescent pregnancy. ^16^ Thus, among adolescents with a greater concern for avoiding unwanted pregnancy, it was reasonable that there is greater interest in knowing the dates of the cycle when there is a greater probability of becoming pregnant. ^43^

### Implications for public health

Adolescent pregnancy is one of the main public health problems among the adolescent and young adult population in Peru. ^6^ The findings of the present study suggest the impact of different individual, sociodemographic and cultural factors on a higher prevalence of adolescent pregnancy. The sociocultural and economic context in Peru determines a high unmet demand for family planning, lack of access to contraception and a low level of knowledge about risky sexual behavior. ^6^^,^^44^

The usefulness of applying prevention policies in other countries to reduce the prevalence of adolescent pregnancy has been described. ^45^^,^^46^ In this regard, Peru has established the Multisectoral Plan for the Prevention of Adolescent Pregnancy 2012-2021, ^9^ which aims to guide the actions of the public sector, civil society and international cooperation agencies in the prevention of adolescent pregnancy, with emphasis on the most vulnerable and poorest groups. Likewise, the Technical Health Standard on Family Planning ^47^ promotes comprehensive care with emphasis on sexual and reproductive health, with the aim of achieving the promotion and the access to contraceptive methods in differentiated schedules and exclusive environments for adolescents.

Therefore, it is important to expand family planning services in order to have an adolescent population informed about sexual and reproductive health with access to traditional or modern contraceptive methods. Likewise, a constant evaluation of the success of these interventions should be reported annually to identify whether there is an improvement or not in the indicators of adolescent pregnancy.

### Strengths and limitations

Although our results are consistent with those reported in previous studies, ^10^^–^^12^^,^^20^ the following limitations should be considered in the present study: First, it should be recognized that because of the cross-sectional design of the study, the associations reported do not imply causality due to the lack of temporality. Second, there may have been recall bias or inadequate understanding of the questions in some subgroups. Third, since the data evaluated came from a secondary database, some variables or risk factors of interest for gestation in adolescents were not included in the measurements made by the ENDES. Despite the above, the ENDES is a nationally and regionally representative survey that has quality control processes and is widely used for the study of health issues in the Peruvian population. For the present study, only data from adolescents between 15 and 19 years of age who initiated sexual relations were included, given that the history of having initiated sexual relations is the causal factor in the existence of pregnancies, thus providing a closer and more homogeneous measurement of the factors associated with adolescent pregnancy, compared to previous reports that evaluated adolescents in general.

## Conclusions

Between 2015 to 2019, in Peru about a third of adolescents aged 15 to 19 years who initiated sexual activity, presented with at least one pregnancy. We identified that being between 17 and 19 years old, having a partner and being of Quechua ethnicity were independently associated with a higher prevalence of adolescent pregnancy. On the other hand, having an occupation, being in school, belonging to the second, third, fourth and fifth quintiles of poverty, having had their first sexual intercourse between 17 and 19 years of age, perceiving a future pregnancy as non-problematic and knowing the moment in the cycle when they could become pregnant were independently associated with a lower prevalence of adolescent pregnancy. It is necessary that the sustained increase of local and national strategies regarding family planning and sexual education in adolescents be carried out in a timely and inclusive manner, given that the avoidance of early initiation of sexual relations together with the acquisition of competencies on adolescent pregnancy prior to the initiation of sexual relations is a reasonable option to reduce the prevalence of adolescent pregnancy and therefore potential obstetric-neonatal complications in Peru.

## Data availability

### Source data

Data used in this study are from the secondary dataset of the Peruvian Demographic and Family Health Surveys - ENDES (2015-2019), available from the “ *El Instituto nacional de Estadística e Informática*” website ( http://iinei.inei.gob.pe/microdatos/). The dataset modules used were: Basic data of women at childbearing age (“ *Datos Basicos de MEF*”); Birth story (“ *Historia de Nacimiento - Tabla de Conocimiento de Metodo*”); Pregnancy, Childbirth, Puerperium and Lactation (“ *Embarazo, Parto, Puerperio y Lactancia*”); and Fertility and partner (“ *Nupcialidad - Fecundidad - Cónyugue y Mujer*”).
