# Prevalence and factors associated with adolescent pregnancy among sexually active adolescent girls in Peru: Evidence from Demographic and Family Health Survey, 2015-2019

**DOI:** 10.12688/f1000research.108837.2

**Published:** 2023-10-02

**Authors:** Brenda Caira-Chuquineyra, Daniel Fernandez-Guzman, Adria Meza-Gómez, Beatriz Milagros Luque-Mamani, Shawny Luz Medina-Carpio, Carlos S. Mamani-García, Marilia Romani-Peña, Cristian Díaz-Vélez

**Affiliations:** 1 Facultad de Medicina, Universidad Nacional de San Agustin de Arequipa, Arequipa, Arequipa, 04001, Peru; 2 Escuela Profesional de Medicina Humana, Universidad Nacional de San Antonio Abad del Cusco, Cusco, 0800, Peru; 3 Universidad Privada Antenor Orrego, Trujillo, 13007, Peru; 4 Instituto de Evaluación de Tecnologías en Salud e Investigación, Lima, Lima, 15001, Peru

**Keywords:** Adolescents, pregnant women, pregnancy in adolescence, pregnancy and motherhood, Peru

## Abstract

**Background:**

The objective of this study was to estimate the prevalence of adolescent pregnancy among sexually active adolescents, and identify the factors associated with this problem.

**Methods:**

A population-based analytical cross-sectional study was conducted using pooled data from the Demographic and Family Health Surveys of Peru, 2015-2019. A total sample of 8850 adolescent girls aged 12 to 19 years who reported a history of sexual intercourse were included. To identify factors related to adolescent pregnancy, the study employed adjusted prevalence ratios (aPR) with 95% confidence intervals (95% CI). The aPR were obtained from a multivariable logistic regression model.

**Results:**

The prevalence of adolescent pregnancy among sexually active adolescents in Peru was 30.9% (95%CI: 29.4–32.4%). In the multivariable analysis; being 17-19 years (aPR: 1.48; 95%CI:1.33–1.64), being married or cohabitant (aPR: 4.01; 95%CI: 3.48–4.61) and belonging to the Quechua ethnicity group (aPR: 1.16; 95%CI: 1.07–1.25), were associated with a higher prevalence. Conversely, the following factors were associated with a lower prevalence of pregnancy: being employed (aPR: 0.81; 95%CI: 0.76–0.86), being currently studying (aPR: 0.43; 95%CI: 0.38–0.49), belonging to the second (aPR: 0.91; 95%CI: 0.85–0.97), third (aPR: 0.81; 95%CI: 0.74–0.89), fourth (aPR: 0.79; 95%CI: 0.69–0.91) and fifth (aPR: 0.59; 95%CI: 0.47–0.75) wealth quintile, initiating sexual relations in middle adolescente (aPR: 0.76; 95%CI: 0.69–0.83) or late adolescence (aPR: 0.40; 95%CI: 0.35–0.46), perceiving a future pregnancy as a problem (aPR: 0.77; 95%CI: 0.72–0.83) and having knowledge of the moment in the cycle when pregnancy can occur (aPR: 0.84; 95%CI: 0.77–0.92)

**Conclusions:**

Approximately three out of ten adolescents who initiated a sexual life had at least one pregnancy. Age, marital status, employment, education, wealth, ethnicity, age at first intercourse, knowledge of when in the cycle she may become pregnant, and perception of future pregnancy were associated with adolescent pregnancy. To decrease the prevalence of teenage pregnancy in Peru, it is imperative to enhance national policies concerning family planning and provide comprehensive sex education targeted at adolescents.

## Introduction

The World Health Organization (WHO) defines adolescents as those between 10 and 19 years of age. ^1^ Adolescence is a critical period characterized by physical, sexual, mental and social growth, which is essential for attaining maturity. ^2^ However, it is during middle (14 to 16 years) and late adolescence (17 to 19 years) stages that adolescents experience increased decision-making responsibility and strive for greater autonomy. ^3^ Consequently, when pregnancy occurs during this period, it can present various challenges for both the adolescent and the infant. ^4^ Delayed prenatal care, increased obstetric and perinatal complications, ^5^ and a higher incidence of maternal mortality are common consequences of adolescent pregnancy. ^6^

According to the WHO, approximately 12 million women between the ages of 15 and 19, and around 1 million girls under the age of 15, give birth each year. ^7^ Latin America and the Caribbean have the second highest rate of adolescent fertility worldwide. Although the overall rate has decreased from 65.6% (2010-2015) to 60.7% (2015-2020), significant variations persist among sub regions and countries. ^8^ In Peru, the Demographic and Family Health Survey (ENDES, for its Spanish acronym) showed that the percentage of adolescents aged 15 to 19 years who were already mothers or were pregnant for the first time remained relatively stable from 2014 to 2019, with a prevalence of 14.6% and 12.6%, respectively. ^9^ Also, adolescent pregnancy has been observed at a higher proportion in women with low educational levels, who reside in rural areas, belong to low socioeconomic strata and according to ethnicity. ^10^ ^–^ ^12^

Although in Peru, different factors associated with adolescent pregnancy have also been reported in small studies or in gray literature, ^13^ ^,^ ^14^ it is not known which factors are associated with adolescent pregnancy. Furthermore, there is a lack of evidence in the existing literature regarding the factors associated with adolescent pregnancy when considering only those adolescents who have initiated sexual relations. This could lead to overestimation of the effects found in other studies. ^10^ ^–^ ^12^ Therefore, the objective of the present study was to estimate the prevalence of adolescent pregnancy among sexually active adolescents, and identify the factors associated with this problem. The identification of associated factors with adolescent pregnancy could be an input for the strengthening of policies on reproductive education and prenatal care.

## Methods

### Study design

For our study, we conducted a secondary analysis of the 2015-2019 ENDES database, which was collected by the National Institute of Statistics and Informatics (INEI, for its Spanish acronym) in Peru. The ENDES survey is a national survey that focuses on private households and their members, including women aged 15-49 years (starting from 2018, women aged 12 years and older were included), children under 5 years and one person aged 15 years and older per household.

Our study utilized an observational study design, specifically an analytical cross-sectional approach. We extracted relevant information from sections of the Women's Individual Questionnaire, which covered demographic and social characteristics, reproductive history, contraceptive methods usage, pregnancy and breastfeeding, nuptiality, fertility preferences, spouse's background, and women's work. These sections provided the necessary data for our analysis.

### Ethical aspects

This study did not require the approval of an ethics committee because the ENDES database is in the public domain and does not allow identification of the subjects, which maintains the corresponding confidentiality. The primary data collection was carried out with the prior signed consent of the interviewees. In addition, the present research project was registered in the “ *Plataforma de Proyectos de Investigación en Salud*” (PRISA) of “ *Instituto Nacional de Salud*” (INS) in Peru with code EI00000001763.

### Population, sample and sampling

The ENDES is a survey with annual representativeness at the national, urban-rural level, by geographic domain and for the 25 departments of Peru. The sampling design of ENDES is two-stage, probabilistic by clusters and stratified at the departmental level and by urban and rural area. The primary sampling unit was made up of the selected clusters. The secondary sampling unit was made up of the selected homes. ^15^

It is important to highlight that the DHS implemented a face-to-face survey methodology, specifically targeting women of reproductive age (15 to 49 years in the 2015, 2016, and 2017 DHS, and 12 to 49 years in the 2018 to 2019 DHS). Within the scope of the ENDES, the survey of women in this age group was carried out in a personalized, confidential, and respectful manner, without requiring the presence of parents. Moreover, it is worth noting that participants who chose not to answer a particular question were recorded as having missing data. This approach aimed to ensure the privacy and comfort of the respondents, fostering an environment where they could provide accurate and honest responses. ^15^

**Figure 1.**
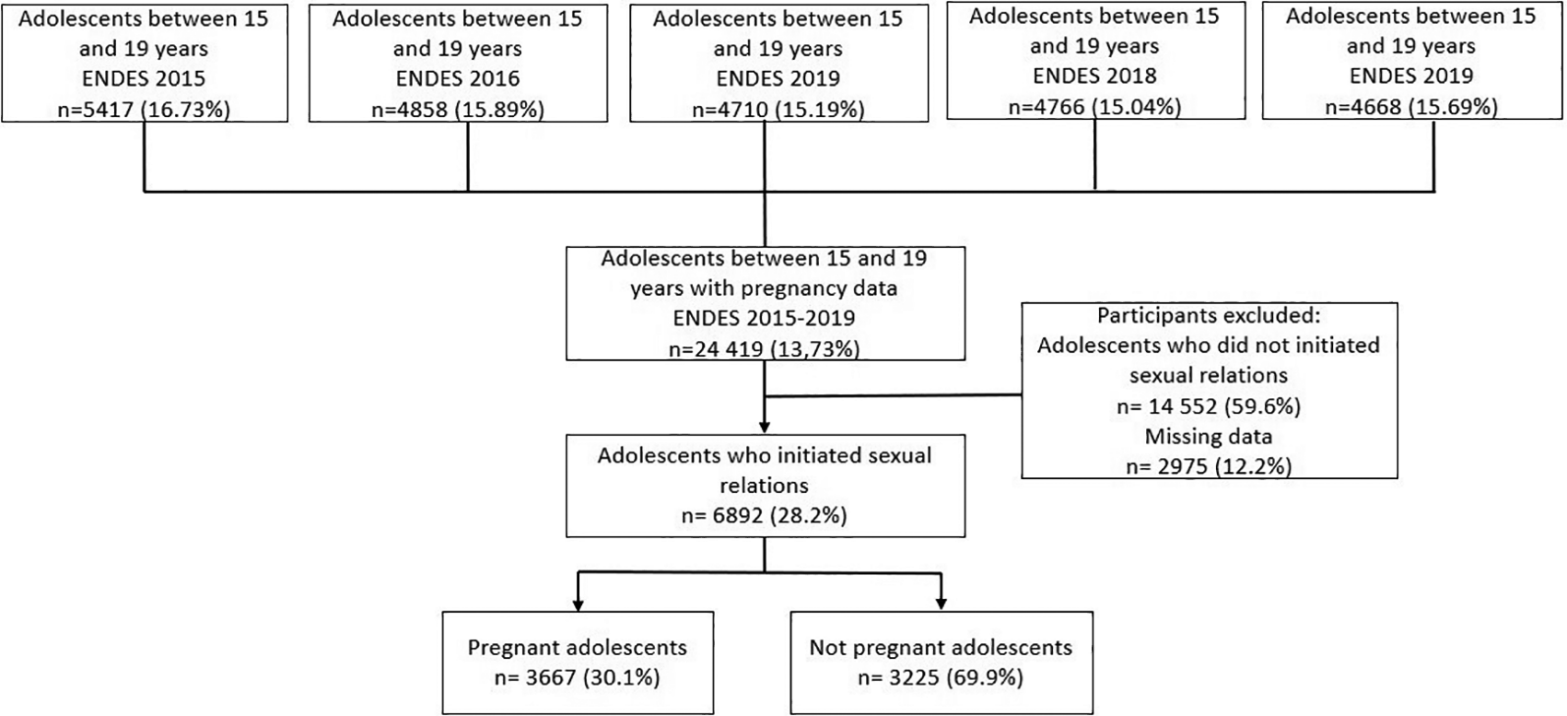
Flow chart of selection of the study sample. ENDES (Spanish acronym), Demographic and Family Health Survey.

During the period of 2015-2019, a total of 31,858 adolescent women between the ages of 12 and 19 were included in the survey. However, for the purpose of our analysis, the effective sample consisted of 8850 women met the criteria of being currently pregnant or already mothers and reported having initiated sexual intercourse. Additionally, those respondents with incomplete information on any of the covariates of interest were excluded ( Figure 1).

### Dependent variable

In our study, adolescent pregnancy served as the dependent variable, and it was assessed through self-reporting using the Individual Woman Questionnaire of the ENDES 2015-2019 survey. The questionnaire included the following questions: “Are you currently pregnant?” This question was coded as V213 in the database, with response options of “No or not sure” or “Yes.”, and the total number of children born. This question was coded as V201 in the database and captured as a numerical variable. It was considered positive if the total number of children born was greater than or equal to 1. These questions were used to collect information about the occurrence of adolescent pregnancy among the participants in our study.

### Independent variables

The following independent variables were considered: Sociodemographic variables, such as age (early and middle adolescence [12 to 16 years], late adolescence [17 to 19 years]), marital status (single, and married or cohabitant), educational level (primary or lower, secondary, and higher education), current occupation (not employed, and employed), currently studying (no, and yes), region (Lima metropolitan, rest of cost, highlands, and jungle), residence area (urban, and rural), wealth index (first quintile [lowest], second quintile, third quintile, fourth quintile, fifth quintile [highest]), and ethnicity (Mestizo, Quechua, negro/moreno/zambo, and other). It is important to note that for the years 2015 and 2016, the ENDES survey did not specifically collect information on ethnicity. As a result, in this study, an alternative approach was taken to approximate ethnicity by considering the information on mother tongue. Specifically, individuals who reported that their mothers spoke Spanish were categorized as mestizos. Those who reported speaking the Quechua language were categorized as Quechua. Individuals who reported other languages were classified under the category of other ethnicities. It is important to acknowledge this limitation when interpreting the results related to ethnicity in the study.

On the other hand, we also consider gynecology-obstetric variables, such as age of first sexual intercourse (early adolescence [10 to 13 years], middle adolescence [14 to 16 years], and late adolescence [17 to 19 years]), use of contraceptive methods (no, traditional contraceptive methods, and modern contraceptive methods), knowledge of fertile period (no, and yes), and perception of future pregnancy (problematic, not problematic). The variable “knowledge of fertile period” was categorized into two groups: those who did not have knowledge of when pregnancy could occur during the menstrual cycle (no) and those who had knowledge of the fertile period (yes). The variable “perception of future pregnancy” was categorized into two groups: those who perceived a future pregnancy as problematic and those who did not perceive it as problematic.

The selection and inclusion of these independent variables in the study was based on a review of the literature. ^10^ ^–^ ^12^

### Statistical analysis

The 2015-2019 ENDES databases were downloaded and imported into Stata ^®^ v.16.0 software (Stata Corporation, College Station, Texas, USA) (Stata, RRID:SCR_012763). All analyses were performed considering the complex sampling design for ENDES using the svy module.

For the descriptive analysis of categorical variables, absolute frequencies and weighted proportions were calculated, and for numerical variables, means with standard deviation were calculated. For bivariate analysis, the association between categorical variables was evaluated using the chi-square test. A value of p<0.05 was considered statistically significant.

To evaluate the association of interest, generalized linear models of the Poisson family with logarithmic link function were used, and we calculated crude prevalence ratios (cPR) and adjusted prevalence ratios (aPR) with their respective 95% confidence intervals (95% CI). For the adjusted model, the method of forward manual selection and the Wald test were used to select the variables to obtain a final parsimonious model. In this way, the variables, including age, marital status, current occupation, currently studying, wealth index, ethnicity, age at first sexual intercourse, knowledge of fertile period and perception of future pregnancy were entered into the final model. Furthermore, to examine the possible role of the area of residence as a modifier of the effect, the adjusted model was analyzed stratified into urban and rural areas. In all analyses conducted in the study, a significance level of p<0.05 was considered to determine statistical significance.

To assess collinearity, the variance inflation factor (VIF) was used, where a value >10 determined multicollinearities between variables; however, all values obtained were less than 10.

Finally, considering a sample size of 8850 respondents and a confidence level of 95%, we assessed the statistical power for each factor analyzed in this study. The statistical power, which indicates the likelihood of detecting a true association, was found to be greater than 80% for all the associations presented. This demonstrates that the sample size included in this study was adequate to detect significant associations between the variables analyzed.

## Results

Out of the total of 31,858 adolescent women aged 12 to 19 years included in the study period, information on the initiation of sexual intercourse was available for 24,466 individuals. Among them, 14,520 were excluded from the analysis because they were not at risk of becoming pregnant due to having no history of sexual intercourse. Additionally, 1,096 individuals were excluded due to missing data in some variables. As a result, the final study population consisted of 8,850 participants who met the criteria and had complete data for the analysis ( Figure 1).

The highest percentage of the study population corresponded to late adolescence (17 to 19 years) (83.1%), single (67.2%), those with secondary (71.1%), those currently in an occupation (58.1%), those belonging to the second wealth quintile (22.6%), and those belonging to the mestizo ethnic group (66.5%), to Lima metropolitan (34.8%) and to an urban area (78.4%) ( Table 1).

**Table 1.** General characteristics of a subsample of Peruvian adolescent women, ENDES 2015-2019 (n=8850).

Characteristics	N	% *	95% CI *
**Age**			
Early and middle adolescence (12 to 16 years)	1624	16.9	15.4-18.5
Late adolescence (17 to 19 years)	7226	83.1	81.5-84.6
**Marital status**			
Single	4477	67.2	65.5-68.9
Married or cohabitant	4373	32.8	31.1-34.5
**Educational level**			
Primary or lower	1043	8.8	7.9-9.8
Secondary	6646	71.1	69.1-73.0
Higher education	1161	20.1	18.3-22.1
**Current occupation**			
Not employed	4025	41.9	39.8-44.0
Employed	4825	58.1	56.0-60.2
**Currently studying**			
No	5398	50.1	48.0-52.1
Yes	3452	49.9	47.9-52.0
**Region**			
Lima metropolitan (Peru's national capital)	1002	34.8	32.2-37.6
Coast (except Lima)	2379	23.2	21.5-24.9
Highlands	2499	21.2	19.6-22.8
Jungle	2970	20.9	19.3-22.6
**Residence area**			
Urban	5834	78.4	77.0-79.6
Rural	3016	21.6	20.4-23.0
**Wealth index**			
First quintile (lowest)	2873	20.4	19.2-21.8
Second quintile	2480	22.6	21.0-24.2
Third quintile	1686	21.5	19.8-23.3
Fourth quintile	1123	19.2	17.3-21.2
Fifth quintile (highest)	688	16.3	14.4-18.4
**Ethnicity**			
Mestizo	5244	66.5	64.5-68.4
Quechua	1750	14.0	12.8-15.3
Negro/moreno/zambo	573	6.3	5.4-7.2
Other	1283	13.3	11.9-14.8
**Age at first sexual intercourse**			
Early adolescence (10 to 13 years)	952	7.3	6.6-8.2
Middle adolescence (14 to 16 years)	5736	61.1	59.1-63.0
Late adolescence (17 to 19 years)	2162	31.6	29.7-33.5
**Use of contraceptive methods**			
No	3694	46.0	43.9-48.2
Traditional contraceptive methods	1049	11.8	10.6-13.0
Modern contraceptive methods	4107	42.2	40.1-44.3
**Knowledge of fertile period**			
No	6673	68.9	66.8-70.9
Yes	2177	31.1	29.1-33.2
**Perception of future pregnancy**			
Problematic	6781	79.4	77.8-80.8
Not problematic	2069	20.6	19.2-22.2
**History of adolescent pregnancy**			
No	4067	69.1	67.6-70.6
Yes	4783	30.9	29.4-32.4

* Weighted values according to complex sampling of the survey.

The prevalence of pregnancy among adolescents aged 15 to 19 years who initiated sexual relations was 30.9%. Several variables were significantly associated with a higher prevalence of pregnancy in this population. Among them, age played a role, with adolescents aged 17 to 19 years having a higher proportion of pregnancies (32.7%; p<0.001). Additionally, having a partner was associated with a higher prevalence of pregnancy (74.3%; p<0.001). Education also played an important role, as adolescents with primary education or lower had a higher proportion of pregnancies (67.3%; p<0.001). Other factors such as unemployment (36.0%; p<0.001), lack of current enrollment in education (52.6%; p<0.001), residing in rural areas (56.2%; p<0.001), belonging to the highlands or jungle region (40.9% and 40.0% respectively; p<0.001), and belonging to the first wealth quintile (56.7%; p<0.001) were also significantly associated with a higher prevalence of pregnancy ( Table 2).

**Table 2.** Prevalence of adolescent pregnancy according to the characteristics of the study population (n=8850).

Characteristics	History of adolescent pregnancy						p-value
	Yes			No			
	n	%	95% CI *	n	%	95% CI *	
**Age**							
Early and middle adolescence (12 to 16 years)	1014	77.9	75.0-80.5	610	22.1	19.5-25.0	**<0.001**
Late adolescence (17 to 19 years)	3053	67.3	65.6-69.0	4173	32.7	31.0-34.4	
**Marital status**							
Single	3490	90.2	89.2-91.2	987	9.8	8.8-10.8	**<0.001**
Married or cohabitant	577	25.7	23.1-28.5	3796	74.3	71.5-76.9	
**Educational level**							
Primary or lower	210	32.7	27.6-38.3	833	67.3	61.7-72.4	**<0.001**
Secondary	2999	67.4	65.6-69.1	3647	32.6	30.9-34.4	
Higher education	858	91.0	89.2-92.6	303	9.0	7.4-10.8	
**Current occupation**							
Not employed	1661	64.0	61.5-66.5	2364	36.0	33.5-38.5	**<0.001**
Employed	2406	72.7	70.8-74.5	2419	27.3	25.5-29.2	
**Currently studying**							
No	1437	47.4	44.8-50.0	3961	52.6	50.0-55.2	**<0.001**
Yes	2630	90.9	89.8-91.8	822	9.1	8.2-10.2	
**Region**							
Lima metropolitan (Peru's national capital)	626	82.0	79.3-84.4	376	18.0	15.6-20.7	**<0.001**
Coast (except Lima)	1127	67.0	64.4-69.5	1252	33.0	30.5-35.6	
Highlands	959	59.1	56.3-61.8	1540	40.9	38.2-43.7	
Jungle	1355	60.0	57.3-62.6	1615	40.0	37.4-42.7	
**Residence area**							
Urban	3098	76.1	74.5-77.6	2736	23.9	22.4-25.5	**<0.001**
Rural	969	43.8	41.3-46.4	2047	56.2	53.6-58.7	
**Wealth index**							
First quintile (lowest)	886	43.3	40.8-45.9	1987	56.7	54.1-59.2	**<0.001**
Second quintile	1075	61.2	58.1-64.2	1405	38.8	35.8-41.9	
Third quintile	896	75.2	72.3-78.0	790	24.8	22.0-27.7	
Fourth quintile	699	81.4	78.4-84.1	424	18.6	15.9-21.6	
Fifth quintile (highest)	511	89.7	87.3-91.6	177	10.3	8.4-12.7	
**Ethnicity**							
Mestizo	2618	72.0	70.2-73.7	2626	28.0	26.3-29.8	**<0.001**
Quechua	649	59.9	55.8-63.9	1101	40.1	36.1-44.2	
Negro/moreno/zambo	248	64.8	59.1-70.1	325	35.2	29.9-40.9	
Other	552	66.1	61.6-70.3	731	33.9	29.7-38.4	
**Age at first sexual intercourse**							
Early adolescence (10 to 13 years)	177	28.8	23.6-34.7	775	71.2	65.3-76.4	**<0.001**
Middle adolescence (14 to 16 years)	2351	64.5	62.4-66.5	3385	35.5	33.5-37.6	
Late adolescence (17 to 19 years)	1539	87.4	85.8-88.8	623	12.6	11.2-14.2	
**Use of contraceptive methods**							
No	2280	80.8	79.1-82.4	1414	19.2	17.6-20.9	**<0.001**
Traditional contraceptive methods	490	67.4	63.2-71.4	559	32.6	28.6-36.8	
Modern contraceptive methods	1297	56.7	54.0-59.4	2810	43.3	40.6-46.0	
**Knowledge of fertile period**							
No	2798	63.8	61.9-65.6	3875	36.2	34.4-38.1	**<0.001**
Yes	1269	80.8	78.7-82.7	908	19.2	17.3-21.3	
**Perception of future pregnancy**							
Problematic	3247	71.5	69.8-73.1	3534	28.5	26.9-30.2	**<0.001**
Not problematic	820	60.0	56.7-63.2	1249	40.0	36.8-43.3	

* Percentages weighted according to complex survey sampling.

Furthermore, the prevalence of adolescent pregnancy was higher among those who initiated their first sexual intercourse between 10 and 13 years (71.2.0%; p<0.001), those who used modern contraceptive methods (43.3%; p<0.001), individuals who did not know the time of the cycle when they could become pregnant (36.2%; p<0.001) and those who did not perceive future pregnancy as a problem (40.0%; p<0.001) ( Table 2).

In the multivariable analysis, several factors were found to be associated with adolescent pregnancy. Being between 17-19 years old (aPR: 1.48; 95%CI: 1.33–1.64), being married or cohabitant (aPR: 4.01; 95%CI: 3.48–4.61), and belonging to the Quechua ethnicity group (aPR: 1.16; 95%CI: 1.07–1.25) were associated with a higher prevalence. Conversely, several factors were associated with a lower prevalence of pregnancy. These included being employed (aPR: 0.81; 95%CI: 0.76–0.86), currently studying (aPR: 0.43; 95%CI: 0.38–0.49), belonging to the second (aPR: 0.91; 95%CI: 0.85–0.97), third (aPR: 0.81; 95%CI: 0.74–0.89), fourth (aPR: 0.79; 95%CI: 0.69–0.91), and fifth (aPR: 0.59; 95%CI: 0.47–0.75) wealth quintile. Additionally, initiating sexual relations in middle adolescence (aPR: 0.76; 95%CI: 0.69–0.83) or late adolescence (aPR: 0.40; 95%CI: 0.35–0.46), perceiving a future pregnancy as a problem (aPR: 0.77; 95%CI: 0.72–0.83), and having knowledge of the moment in the cycle when pregnancy can occur (aPR: 0.84; 95%CI: 0.77–0.92) were associated with a lower prevalence of pregnancy ( Table 3).

**Table 3.** Factors associated with adolescent pregnancy.

Characteristics	Crude model			Parsimonious adjusted model		
	cPR	95% CI	p-value	aPR	95% CI	p-value
**Age**						
Early and middle adolescence (12 to 16 years)	Ref.			Ref.		
Late adolescence (17 to 19 years)	1.48	1.29-1.69	**<0.001**	1.48	1.33-1.64	**<0.001**
**Marital status**						
Single	Ref.			Ref.		
Married or cohabitant	7.61	6.79-8.53	**<0.001**	4.01	3.48-4.61	**<0.001**
**Educational level**						
Primary or lower	Ref.					
Secondary	0.48	0.44-0.53	**<0.001**			
Higher education	0.13	0.11-0.16	**<0.001**			
**Current occupation**						
Not employed	Ref.			Ref.		
Employed	0.76	0.69-0.84	**<0.001**	0.81	0.76-0.86	**<0.001**
**Currently studying**						
No	Ref.			Ref.		
Yes	0.17	0.15-0.20	**<0.001**	0.43	0.38-0.49	**<0.001**
**Region**						
Lima metropolitan (Peru's national capital)						
Coast (except Lima)	1.83	1.55-2.17	**<0.001**			
Highlands	2.27	1.93-2.67	**<0.001**			
Jungle	2.22	1.89-2.61	**<0.001**			
**Residence area**						
Urban	Ref.					
Rural	2.35	2.17-2.54	**<0.001**			
**Wealth index**						
First quintile (lowest)	Ref.			Ref.		
Second quintile	0.68	0.62-0.75	**<0.001**	0.91	0.85-0.97	**0.003**
Third quintile	0.44	0.39-0.50	**<0.001**	0.81	0.74-0.89	**<0.001**
Fourth quintile	0.33	0.28-0.39	**<0.001**	0.79	0.69-0.91	**0.001**
Fifth quintile (highest)	0.18	0.15-0.23	**<0.001**	0.59	0.47-0.75	**<0.001**
**Ethnicity**						
Mestizo	Ref.			Ref.		
Quechua	1.43	1.27-1.62	**<0.001**	1.16	1.07-1.25	**<0.001**
Negro/moreno/zambo	1.26	1.06-1.50	**0.011**	0.98	0.88-1.10	0.784
Other	1.21	1.05-1.40	**0.010**	0.94	0.85-1.03	0.164
**Age at first sexual intercourse**						
Early adolescence (10 to 13 years)	Ref.			Ref.		
Middle adolescence (14 to 16 years)	0.50	0.45-0.55	**<0.001**	0.76	0.69-0.83	**<0.001**
Late adolescence (17 to 19 years)	0.18	0.15-0.21	**<0.001**	0.40	0.35-0.46	**<0.001**
**Use of contraceptive methods**						
No	Ref.					
Traditional contraceptive methods	1.70	1.45-1.99	**<0.001**			
Modern contraceptive methods	2.26	2.03-2.51	**<0.001**			
**Knowledge of fertile period**						
No	Ref.			Ref.		
Yes	0.53	0.47-0.60	**<0.001**	0.84	0.77-0.92	**<0.001**
**Perception of future pregnancy**						
Problematic	Ref.			Ref.		
Not problematic	1.40	1.26-1.55	**<0.001**	0.77	0.72-0.83	**<0.001**

cPR: crude prevalence ratio; aPR: adjusted prevalence ratio; 95% CI: 95% confidence interval.

Prevalence ratio and confidence intervals were calculated considering complex survey sampling. The p-values <0.05 are in bold.

Factors associated with adolescent pregnancy in urban and rural areas are presented in Table 4 and Table 5, respectively.

**Table 4.** Factors associated with adolescent pregnancy in urban areas.

Characteristics	Crude model			Parsimonious adjusted model		
	cPR	95% CI	p-value	aPR	95% CI	p-value
**Age**						
Early and middle adolescence (12 to 16 years)	Ref.			Ref.		
Late adolescence (17 to 19 years)	1.53	1.26-1.86	**<0.001**	1.47	1.24-1.73	**<0.001**
**Marital status**						
Single	Ref.			Ref.		
Married or cohabitant	9.38	8.04-10.93	**<0.001**	5.15	4.25-6.24	**<0.001**
**Educational level**						
Primary or lower	Ref.					
Secondary	0.49	0.40-0.59	**<0.001**			
Higher education	0.15	0.11-0.20	**<0.001**			
**Current occupation**						
Not employed	Ref.			Ref.		
Employed	0.75	0.65-0.85	**<0.001**	0.79	0.72-0.87	**<0.001**
**Currently studying**						
No	Ref.			Ref.		
Yes	0.17	0.15-0.20	**<0.001**	0.43	0.36-0.51	**<0.001**
**Region**						
Lima metropolitan (Peru's national capital)	Ref.					
Coast (except Lima)	1.64	1.39-1.95	**<0.001**			
Highlands	1.53	1.27-1.84	**<0.001**			
Jungle	1.57	1.32-1.87	**<0.001**			
**Wealth index**						
First quintile	Ref.			Ref.		
Second quintile	0.75	0.64-0.88	**<0.001**	0.93	0.82-1.05	0.227
Third quintile	0.50	0.42-0.60	**<0.001**	0.86	0.75-0.98	**0.024**
Fourth quintile	0.38	0.31-0.47	**<0.001**	0.87	0.74-1.02	0.096
Fifth quintile	0.21	0.17-0.28	**<0.001**	0.68	0.53-0.88	**0.003**
**Ethnicity**						
Mestizo	Ref.			Ref.		
Quechua	1.30	1.06-1.60	**0.013**	1.18	1.04-1.35	**0.011**
Negro/moreno/zambo	1.27	1.01-1.59	**0.040**	0.93	0.81-1.08	0.349
Other	1.05	0.84-1.30	0.694	0.89	0.76-1.04	0.144
**Age at first sexual intercourse**						
Early adolescence (10 to 13 years)	Ref.			Ref.		
Middle adolescence (14 to 16 years)	0.45	0.39-0.53	**<0.001**	0.71	0.61-0.82	**<0.001**
Late adolescence (17 to 19 years)	0.14	0.11-0.17	**<0.001**	0.35	0.28-0.43	**<0.001**
**Use of contraceptive methods**						
No	Ref.					
Traditional contraceptive methods	1.77	1.40-2.23	**<0.001**			
Modern contraceptive methods	2.54	2.20-2.93	**<0.001**			
**Knowledge of fertile period**						
No	Ref.			Ref.		
Yes	0.56	0.48-0.65	**<0.001**	0.86	0.76-0.97	**0.011**
**Perception of future pregnancy**						
Problematic	Ref.			Ref.		
Not problematic	1.37	1.18-1.59	**<0.001**	0.76	0.68-0.86	**<0.001**

cPR: crude prevalence ratio; aPR: adjusted prevalence ratio; 95% CI: 95% confidence interval.

Prevalence ratio and confidence intervals were calculated considering complex survey sampling. The p-values <0.05 are in bold.

**Table 5.** Factors associated with adolescent pregnancy in rural areas.

Characteristics	Crude model			Parsimonious adjusted model		
	cPR	95% CI	p-value	aPR	95% CI	p-value
**Age**						
Early and middle adolescence (12 to 16 years)	Ref.			Ref.		
Late adolescence (17 to 19 years)	1.72	1.51-1.96	**<0.001**	1.54	1.38-1.71	**<0.001**
**Marital status**						
Single	Ref.			Ref.		
Married or cohabitant	3.30	2.90-3.74	**<0.001**	2.17	1.91-2.45	**<0.001**
**Educational level**						
Primary or lower	Ref.					
Secondary	0.69	0.63-0.75	**<0.001**			
Higher education	0.26	0.18-0.37	**<0.001**			
**Current occupation**						
Not employed	Ref.			Ref.		
Employed	0.79	0.72-0.86	**<0.001**	0.83	0.78-0.89	**<0.001**
**Currently studying**						
No	Ref.			Ref.		
Yes	0.27	0.23-0.32	**<0.001**	0.47	0.40-0.55	**<0.001**
**Region**						
Coast (except Lima)	Ref.					
Highlands	1.01	0.87-1.16	0.914			
Jungle	1.07	0.92-1.23	0.373			
**Wealth index**						
First quintile	Ref.			Ref.		
Second quintile	0.84	0.75-0.95	**0.005**	0.94	0.86-1.02	0.133
Third quintile	0.79	0.59-1.07	0.126	0.98	0.81-1.19	0.848
Fourth quintile	0.48	0.22-1.06	0.069	0.96	0.56-1.63	0.881
Fifth quintile	0.12	0.01-1.01	0.051	0.35	0.06-1.89	0.220
**Ethnicity**						
Mestizo	Ref.			Ref.		
Quechua	0.99	0.89-1.09	0.799	1.11	1.03-1.19	**0.008**
Negro/moreno/zambo	1.02	0.84-1.24	0.844	1.06	0.91-1.23	0.480
Other	1.00	0.89-1.12	0.947	0.97	0.88-1.05	0.435
**Age first sexual intercourse**						
Early adolescence (10 to 13 years)	Ref.			Ref.		
Middle adolescence (14 to 16 years)	0.70	0.64-0.77	**<0.001**	0.80	0.74-0.86	**<0.001**
Late adolescence (17 to 19 years)	0.39	0.33-0.46	**<0.001**	0.48	0.41-0.55	**<0.001**
**Use of contraceptive methods**						
No	Ref.					
Traditional contraceptive methods	1.24	1.07-1.44	**0.004**			
Modern contraceptive methods	1.78	1.61-1.97	**<0.001**			
**Knowledge of fertile period**						
No	Ref.			Ref.		
Yes	0.67	0.58-0.76	**<0.001**	0.82	0.74-0.91	**<0.001**
**Perception of future pregnancy**						
Problematic	Ref.			Ref.		
Not problematic	1.13	1.03-1.24	**0.008**	0.81	0.75-0.86	**<0.001**

cPR: crude prevalence ratio; aPR: adjusted prevalence ratio; 95% CI: 95% confidence interval.

Prevalence ratio and confidence intervals were calculated considering complex survey sampling. The p-values <0.05 are in bold.

## Discussion

In Peru, about three in 10 adolescents between 15 and 19 years of age who have initiated sexual relations have had at least one pregnancy. The high prevalence of adolescent pregnancy could favor the appearance of a higher rate of obstetric and perinatal complications in this group. ^5^ It was found in the present study that older age (17 to 19 years), the presence of a partner, ethnicity, having a job, being in school, level of wealth, early initiation of sexual relations (≤16 years), the perception of a future pregnancy as non-problematic, and knowledge of the moment in the cycle when pregnancy may occur were independently associated with adolescent pregnancy.

We found that belonging to late adolescence (between 17 to 19 years) was associated with a higher prevalence of adolescent pregnancy. This is consistent with previous studies ^16^ ^,^ ^17^ and could be explained by the greater development and mental maturity in this group to assume a pregnancy, as well as a greater development of female identity and greater capacity to adapt to parenting roles. ^3^ Similarly, the literature has described a greater desire to become pregnant in late adolescence compared to the rest of adolescence. ^18^ Furthermore, it should be taken into account that the sociocultural and economic context could influence the age of onset of risky sexual behaviors in adolescents, ^19^ which could lead to a higher risk of becoming pregnant.

The presence of a partner among the adolescents was associated with a higher prevalence of adolescent pregnancy. In this regard, this association was previously evidenced in studies focusing on sexually active adolescents. ^20^ ^,^ ^21^ In Latin American countries, the high prevalence of adolescent pregnancy could be due to the fact that among adolescents with a partner there is a greater desire to become pregnant and achieve motherhood in order to start a family at an early age. ^22^ In Peru, it has been reported that 69% of adolescents aged 15 to 19 years who were pregnant or had children were in some type of early union (65.8% cohabiting and 3.2% married). ^23^ Thus, the association with the presence of a partner could be due to the fact that after adolescents become pregnant, parents put pressure on the couple to marry or cohabit. Given that, family planning measures should be widely encouraged for both female and male adolescents.

We also found that belonging to the Quechua ethnicity group was associated with a higher prevalence of adolescent pregnancy. In this regard, it has been reported that women from ethnic minorities tend to experience social and economic exclusion, which could generate greater inequity in access to family planning services and contraceptive methods, ^24^ ^,^ ^25^ leading to higher maternal mortality. ^26^ In Peru in 2016, it was observed that the population of native origin (Quechua, Aymara or Amazonian origin) had a higher level of fertility and a lower proportion of contraceptive methods used. ^27^ In these ethnic groups, a greater acceptance of early marriage and pregnancy has also been reported. ^25^ Therefore, greater state intervention is required in these population groups to reduce the gaps in access to sexual and reproductive health information for adolescents.

Having an occupation or being a student was associated with a lower frequency of adolescent pregnancy. Adolescents who engage in these activities may prioritize education or economic income over starting a family. ^28^ This contrasts with previous studies conducted in middle- and low-income countries, where a higher risk of pregnancy has been reported among adolescents who do not have an occupation ^26^ or who do not attend school. ^29^ Having an education could be related to a better knowledge of sexual health and a greater ambition to complete higher education, postponing reproductive desire until greater emotional and economic stability is achieved. ^30^ In Peru, dropout at the secondary education level in 2015 was 7.6% among adolescents. ^31^ Given this, it would be important to implement national programs that promote and ensure education and find vulnerable adolescents who have dropped out of school.

It was also found in the present study that belonging to the third, fourth or fifth wealth quintile was associated with a lower frequency of adolescent pregnancy. In Latin America and the Caribbean, an early onset of sexual relations ^32^ and a higher proportion of adolescent pregnancy ^33^ ^–^ ^35^ were reported among lower income social groups. This could be explained by lower use of and limited access to contraceptive methods in these groups. ^35^ Similarly, unfavorable economic conditions could lead women to think of motherhood as a better life option, since they would have a partner to take care of household needs. ^16^ ^,^ ^17^ In Peru, the “Juntos” Program was implemented in 2005 with the objective of reducing the impact of poverty and its intergenerational transmission through the bimonthly delivery of a monetary incentive of 200 nuevos soles (S/200) to low income households, plus an additional S/100 for pregnant women who attend antenatal visit care and S/100 for each child under 3 years of age who comply with growth and development checkups. ^36^ This initiative could favor vulnerable groups such as pregnant adolescents in low socioeconomic strata.

Regarding the age of initiation of sexual intercourse, we found that late initiation of sexual intercourse (17 to 19 years) was associated with a lower prevalence of adolescent pregnancy. This finding is consistent with the literature, ^20^ which has reported earlier ages of sexual intercourse, ^32^ ^,^ ^37^ and higher proportions of a first pregnancy between 15 and 19 years of age. ^38^ This could be explained by the lack of promotion of sexual and reproductive health information, including family planning methods, at earlier ages. Therefore, the general population should be made aware of the importance of regulating access to sexual and reproductive health information from puberty and adolescence.

Another variable that was associated with a higher prevalence of adolescent pregnancy was the perception of a future pregnancy as non-problematic in the crude analysis. In this regard, it has been previously reported that a positive attitude toward pregnancy among postpartum adolescents is strongly associated with a higher prevalence of a second pregnancy. ^39^ Likewise, a greater likelihood of feeling stigmatized during pregnancy has been observed when they did not have a romantic relationship with a partner or felt verbally abused by family, friends, partners or other adolescents. ^40^ This could be due to the fact that the greater emotional stability achieved through the support of a partner and family, friends and social environment during pregnancy could generate a non-problematic perception of a subsequent pregnancy. ^41^ ^,^ ^42^ However, in our analysis, when adjusting for other variables, we found a reversal in the direction of the association. This could be explained by the fact that the sample evaluated included adolescents with no history of pregnancy, who could have a biased perception of a possible pregnancy and be influenced by the desire to start a family. This explanation is consistent with the fact that there is a high prevalence of desire for pregnancy among adolescents in Latin America. ^22^

Knowledge of the moment in the cycle when pregnancy is possible was associated with a lower prevalence of adolescent pregnancy. This is consistent with previous studies, where ignorance of the fertile days was a risk factor for adolescent pregnancy. ^16^ Thus, among adolescents with a greater concern for avoiding unwanted pregnancy, it was reasonable that there is greater interest in knowing the dates of the cycle when there is a greater probability of becoming pregnant. ^43^

### Implications for public health

Adolescent pregnancy is one of the main public health problems among the adolescent and young adult population in Peru. ^6^ The findings of the present study suggest the impact of different individual, sociodemographic and cultural factors on a higher prevalence of adolescent pregnancy. The sociocultural and economic context in Peru determines a high unmet demand for family planning, lack of access to contraception and a low level of knowledge about risky sexual behavior. ^6^ ^,^ ^44^

The usefulness of applying prevention policies in other countries to reduce the prevalence of adolescent pregnancy has been described. ^45^ ^,^ ^46^ In this regard, Peru has established the Multisectoral Plan for the Prevention of Adolescent Pregnancy 2012-2021, ^9^ which aims to guide the actions of the public sector, civil society and international cooperation agencies in the prevention of adolescent pregnancy, with emphasis on the most vulnerable and poorest groups. Likewise, the Technical Health Standard on Family Planning ^47^ promotes comprehensive care with emphasis on sexual and reproductive health, with the aim of achieving the promotion and the access to contraceptive methods in differentiated schedules and exclusive environments for adolescents.

Therefore, it is important to expand family planning services in order to have an adolescent population informed about sexual and reproductive health with access to traditional or modern contraceptive methods. Likewise, a constant evaluation of the success of these interventions should be reported annually to identify whether there is an improvement or not in the indicators of adolescent pregnancy.

### Strengths and limitations

Although our results are consistent with those reported in previous studies, ^10^ ^–^ ^12^ ^,^ ^20^ the following limitations should be considered in the present study: First, it should be recognized that because of the cross-sectional design of the study, the associations reported do not imply causality due to the lack of temporality. Second, there may have been recall bias or inadequate understanding of the questions in some subgroups. Third, since the data evaluated came from a secondary database, some variables or risk factors of interest for gestation in adolescents were not included in the measurements made by the ENDES. Despite the above, the ENDES is a nationally and regionally representative survey that has quality control processes and is widely used for the study of health issues in the Peruvian population. For the present study, only data from adolescents between 15 and 19 years of age who initiated sexual relations were included, given that the history of having initiated sexual relations is the causal factor in the existence of pregnancies, thus providing a closer and more homogeneous measurement of the factors associated with adolescent pregnancy, compared to previous reports that evaluated adolescents in general.

## Conclusions

Between 2015 to 2019, in Peru about a third of adolescents aged 15 to 19 years who initiated sexual activity, presented with at least one pregnancy. We identified that being between 17 and 19 years old, having a partner and being of Quechua ethnicity were independently associated with a higher prevalence of adolescent pregnancy. On the other hand, having an occupation, being in school, belonging to the second, third, fourth and fifth quintiles of poverty, having had their first sexual intercourse between 17 and 19 years of age, perceiving a future pregnancy as non-problematic and knowing the moment in the cycle when they could become pregnant were independently associated with a lower prevalence of adolescent pregnancy. It is necessary that the sustained increase of local and national strategies regarding family planning and sexual education in adolescents be carried out in a timely and inclusive manner, given that the avoidance of early initiation of sexual relations together with the acquisition of competencies on adolescent pregnancy prior to the initiation of sexual relations is a reasonable option to reduce the prevalence of adolescent pregnancy and therefore potential obstetric-neonatal complications in Peru.

## Data availability

### Source data

Data used in this study are from the secondary dataset of the Peruvian Demographic and Family Health Surveys - ENDES (2015-2019), available from the “ *El Instituto nacional de Estadística e Informática*” website ( http://iinei.inei.gob.pe/microdatos/). The dataset modules used were: Basic data of women at childbearing age (“ *Datos Basicos de MEF*”); Birth story (“ *Historia de Nacimiento - Tabla de Conocimiento de Metodo*”); Pregnancy, Childbirth, Puerperium and Lactation (“ *Embarazo, Parto, Puerperio y Lactancia*”); and Fertility and partner (“ *Nupcialidad - Fecundidad - Cónyugue y Mujer*”).
