# Supplementary material for: Prevalence and factors associated with adolescent pregnancy among sexually active adolescent girls in Peru: Evidence from Demographic and Family Health Survey, 2015-2019
Source: F1000Res. 2023 Oct 2;11:566. Originally published 2022 May 25. [Version 2] doi: 10.12688/f1000research.108837.2 (PMC10958153; doi:10.12688/f1000research.108837.2)
Supplement: Peer review history

# Reviewer report: Reviewer response for version 2

**DOI:** 10.5256/f1000research.151332.r251292

**Published:** 2024-03-21

**Authors:** Olusegun S. Ewemooje

**Affiliations:** 1 University of Botswana, Gaborone, South-East District, Botswana

The document has been improved greatly by addressing earlier reviewers' comments. However, I have the following comments for the authors;

 Major concern

 1. On page 7, Table 2, There is a big problem with Table 2, e.g. under "age - Late adolescence (17-19 years)", YES has n=3053 (61.3%) while NO has n=4173 (32.7%). This needs to be checked because percentage of NO should be greater than that of YES. This same mistake can be found in all the characteristics considered. Therefore, the whole take should be recalculated. 

 Minor concern

 1. Under discussion, paragraph 5. This statement "Adolescents who engage in these activities may prioritize education or economic income over starting a family. This contrasts with previous studies conducted in middle- and low-income countries, where a higher risk of pregnancy has been reported among adolescents who do not have an occupation26 or who do not attend school", is not consistent with the analysis. This do not contradict previous studies but rather support the.

 2. Under Strength and limitations

 "For the present study, only data from adolescents between 15 and 19 years of age who initiated sexual relations were included,..." This limitation should be removed or redefine to capture aged 12-19 years.

Is the work clearly and accurately presented and does it cite the current literature?

Yes

If applicable, is the statistical analysis and its interpretation appropriate?

Partly

Are all the source data underlying the results available to ensure full reproducibility?

Yes

Is the study design appropriate and is the work technically sound?

Yes

Are the conclusions drawn adequately supported by the results?

Yes

Are sufficient details of methods and analysis provided to allow replication by others?

Yes

Reviewer Expertise:

Survey Design & Applications; Population & Health Studies

I confirm that I have read this submission and believe that I have an appropriate level of expertise to confirm that it is of an acceptable scientific standard, however I have significant reservations, as outlined above.

# Reviewer report: Reviewer response for version 2

**DOI:** 10.5256/f1000research.151332.r211361

**Published:** 2023-10-06

**Authors:** Angelo Mark Walag

**Affiliations:** 1 Department of Science Education, University of Science and Technology of Southern Philippines, Cagayan de Oro, Northern Mindanao, Philippines

The present paper can be accepted

Is the work clearly and accurately presented and does it cite the current literature?

Yes

If applicable, is the statistical analysis and its interpretation appropriate?

Yes

Are all the source data underlying the results available to ensure full reproducibility?

Yes

Is the study design appropriate and is the work technically sound?

Yes

Are the conclusions drawn adequately supported by the results?

Yes

Are sufficient details of methods and analysis provided to allow replication by others?

Yes

Reviewer Expertise:

Science and Health Education, Natural Sciences

I confirm that I have read this submission and believe that I have an appropriate level of expertise to confirm that it is of an acceptable scientific standard.

# Reviewer report: Reviewer response for version 1

**DOI:** 10.5256/f1000research.120271.r170294

**Published:** 2023-04-26

**Authors:** Angelo Mark Walag

**Affiliations:** 1 Department of Science Education, University of Science and Technology of Southern Philippines, Cagayan de Oro, Northern Mindanao, Philippines

The paper demonstrates an initial study exploring the prevalence and associated factors of adolescent pregnancy in Peru from 2015-2019. This study will be meaningful to research scholars interested in sociological and behavioral aspects of adolescent pregnancy as well as policymakers interested in the phenomena.

 While the study has interesting results, it needs some revisions. 

1. In the abstract, minimize the presentation of specific results as this is intimidating to readers. Just present key findings of the study. Keywords that already appear in the title need to be replaced to maximize visibility and online reach.
2. The introduction section may be expanded covering literature outside of Peru. The authors may also present some findings related to prevalence of adolescent pregnancy in different parts of the globe. This will help the authors to establish novelty and gap in the literature.
3. It is highly commendable that the researchers discussed ethical consideration of the study.
4. Methods are appropriately and thoroughly discussed
5. The result that there is higher prevalence of adolescent pregnancy in those who use contraceptives need to be discussed further as this is a surprising result. Authors may need to offer possible explanation for this.
6. The rest of the discussion is well-done
7. Conclusion is sufficient
8. References are appropriate.

Is the work clearly and accurately presented and does it cite the current literature?

Yes

If applicable, is the statistical analysis and its interpretation appropriate?

Yes

Are all the source data underlying the results available to ensure full reproducibility?

Yes

Is the study design appropriate and is the work technically sound?

Yes

Are the conclusions drawn adequately supported by the results?

Yes

Are sufficient details of methods and analysis provided to allow replication by others?

Yes

Reviewer Expertise:

Science and Health Education, Natural Sciences

I confirm that I have read this submission and believe that I have an appropriate level of expertise to confirm that it is of an acceptable scientific standard.

# Response

**Published:** 2023-06-16

**Authors:** Brenda Sofia Caira-Chuquineyra

**Affiliations:** Universidad Nacional de San Agustin, Peru

Response #1

 We appreciate your comment.

 We have restructured the results section of the summary to make it clearer. We have also added some keywords

 “The prevalence of adolescent pregnancy among sexually active adolescents in Peru was 30.9% (95%CI: 29.4–32.4%). In the multivariable analysis; being 17-19 years (aPR: 1.48; 95%CI :1.33–1.64), being married or cohabitant (aPR: 4.01; 95%CI: 3.48–4.61) and belonging to the Quechua ethnicity group (aPR: 1.16; 95%CI: 1.07–1.25), were associated with a higher prevalence. Conversely, the following factors were associated with a lower prevalence of pregnancy: being employed (aPR: 0.81; 95%CI: 0.76–0.86), being currently studying (aPR: 0.43; 95%CI: 0.38–0.49), belonging to the second (aPR: 0.91; 95%CI: 0.85–0.97), third (aPR: 0.81; 95%CI: 0.74–0.89), fourth (aPR: 0.79; 95%CI: 0.69–0.91) and fifth (aPR: 0.59; 95%CI: 0.47–0.75) wealth quintile, initiating sexual relations in middle adolescente (aPR: 0.76; 95%CI: 0.69–0.83) or  late adolescence (aPR: 0.40; 95%CI: 0.35–0.46), perceiving a future pregnancy as a problem (aPR: 0.77; 95%CI: 0.72–0.83) and having knowledge of the moment in the cycle when pregnancy can occur (aPR: 0.84; 95%CI: 0.77–0.92)”

 Response #2

 We appreciate your comment.

 In the second paragraph of the introduction, we detailed adolescent pregnancy rates globally and in the Latin American region.

 “According to the WHO, approximately 12 million women between the ages of 15 and 19, and around 1 million girls under the age of 15, give birth each year. 7 Latin America and the Caribbean have the second highest rate of adolescent fertility worldwide. Although the overall rate has decreased from 65.6% (2010-2015) to 60.7% (2015-2020), significant variations persist among sub regions and countries”

 Response #3

 We appreciate your comment.

 The section on "Ethical aspects" was considered as the second subtitle of Methods.

 Response #4

 We appreciate your comment.

 Response #5

 We appreciate your comment.

 While it is true that this finding is surprising. We should point out that temporality for this item could be an important point to consider. Given that the use of these methods refers to a current use, which could be a consequence of having experienced a pregnancy, she began to take care of herself so as not to have more children. On the other hand, in those women with no history of pregnancy, it is possible that they are initiating their sexual life and were not using any method. Despite the implications that this finding could represent, it was not considered in the adjusted analysis and was therefore not a widely discussed point.

 Response #6

 We appreciate your comment.

 Response #7

 We appreciate your comment

 Response #8

 We appreciate your comment.

# Reviewer report: Reviewer response for version 1

**DOI:** 10.5256/f1000research.120271.r140010

**Published:** 2022-06-20

**Authors:** Dora Blitchtein-Winicki

**Affiliations:** 1 School of Medicine, Research and Innovation Center of the Faculty of Health Sciences, Universidad Peruana de Ciencias Aplicadas, Lima, Peru; 2 Universidad de Ciencias Aplicadas, Lima, Peru

This paper explores a relevant public health issue about the factors associated with teenage pregnancy, the resulting information has a significant potential in selecting and directing strategies in for rural and urban adolescent population.

1. **Methodology**
  - **1.1: **It is necessary to specify the selection criteria of the study population, the inclusion and exclusion criteria are not clear or fully described, it is stated in the results section that people with missing data for all the independent variables were excluded, it should be explicit in the section on population.
  - **1.2**: It is necessary to encompass information about the statistical power of the study with the amount of population that met the selection criteria of the study, it is possible to select prevalence of two factors with lower prevalence in the exposed and unexposed population to do so.
  - **1.3: **More details are needed on the basis of which questions and how the independent variables were categorized, for example, how the positive or negative perception of pregnancy, the knowledge of the cycle in which she could become pregnant, and the use of contraceptive methods (any? or modern? in her life, current use?) were categorized. In regard to the latter, the articles cited include the unmet need for modern contraceptive methods as one of the related factors, however, it is not understood in the study whether the knowledge and/or use of modern contraceptive methods has been included as a factor.
  - **1.4**: Please clarify regarding the ethnicity variable from which questions it was taken and how it was classified, the categories presented in the descriptive table called ethnicity that includes mestizo, quecha, negro/moreno/zambo and others, are only included in the Peruvian Demographic Health Survey ENDES survey since 2018 and 2019. The question encompassed from 2015 to 2019 was mother tongue during childhood and the categories were Spanish, Quechua, Aymara, other language. http://iinei.inei.gob.pe/microdatos/
  - **1.5**: Describe the study procedures for data collection of main variables of the present study in the Demographic Health Survey of Peru ENDES, was it a face-to-face interview, were the personnel expressly trained for these modules, are there any particular characteristics related to women aged 15 to 17 years? Or in women in general for the information modules encompassed?
2. **Data Analysis**
  - **2.1**: It is not clear whether multicollinearity was evaluated in the adjusted models.
3. **Results**
  - **3.1:** Figure 1. In the ENDES annual information of participants, 2017 information is missing and 2019 is repeated with different information (n). It is not specified how many were excluded due to missing data for each variable encompassed as factors.
  - **3.2:** It is not understood how 24,419 can have information of pregnancy and of those 14,552 had not initiated sexual intercourse? this needs to be reworded or clearly explained **.**
4. **Discussion**
  - **​​​​​​​4.1** The discussion should include aspects of gender and cultural relations according to ethnicity or context, such as the type of family they come from (no parents, nuclear, extended), age of cohabitation and types of cohabitation

Is the work clearly and accurately presented and does it cite the current literature?

Yes

If applicable, is the statistical analysis and its interpretation appropriate?

Yes

Are all the source data underlying the results available to ensure full reproducibility?

Partly

Is the study design appropriate and is the work technically sound?

Yes

Are the conclusions drawn adequately supported by the results?

Yes

Are sufficient details of methods and analysis provided to allow replication by others?

Partly

Reviewer Expertise:

Public health

I confirm that I have read this submission and believe that I have an appropriate level of expertise to confirm that it is of an acceptable scientific standard, however I have significant reservations, as outlined above.

# Response

**Published:** 2023-06-16

**Authors:** Brenda Sofia Caira-Chuquineyra

**Affiliations:** Universidad Nacional de San Agustin, Peru

Response #1

 We appreciate your comment.

 We have added information on inclusion and exclusion criteria in the population section. 

 “During the period of 2015-2019, a total of 31,858 adolescent women between the ages of 12 and 19 were included in the survey. However, for the purpose of our analysis, the effective sample consisted of 8850 women met the criteria of being currently pregnant or already mothers and reported having initiated sexual intercourse. Additionally, those respondents with incomplete information on any of the covariates of interest were excluded”

 Response #2

 We appreciate your comment.

 We calculated the statistical power for each association presented and added a paragraph noting the results.

 Statistical analysis section: “Finally, considering a sample size of 8850 respondents and a confidence level of 95%, we assessed the statistical power for each factor analyzed in this study. The statistical power, which indicates the likelihood of detecting a true association, was found to be greater than 80% for all the associations presented. This demonstrates that the sample size included in this study was adequate to detect significant associations between the variables analyzed

 ”

 Response #3

 We appreciate your comment.

 We have detailed and used the appropriate terms to differentiate the concept of the variable to be used. 

 For example, for the variable use of contraceptive methods we categorized the different methods used into three groups: no use, use of traditional methods and use of modern methods.

 On the other hand, for the variable knowledge of the fertile period, we have expanded when it corresponded to a response of no and yes respectively. 

 “On the other hand, we also consider gynecology-obstetric variables, such as age of first sexual intercourse (early adolescence [10 to 13 years], middle adolescence [14 to 16 years], and late adolescence [17 to 19 years]), use of contraceptive methods (no, traditional contraceptive methods, and modern contraceptive methods), knowledge of fertile period (no, and yes), and perception of future pregnancy (problematic, not problematic). The variable "knowledge of fertile period" was categorized into two groups: those who did not have knowledge of when pregnancy could occur during the menstrual cycle (no) and those who had knowledge of the fertile period (yes). The variable "perception of future pregnancy" was categorized into two groups: those who perceived a future pregnancy as problematic and those who did not perceive it as problematic.”

 Response #4

 We appreciate your comment.

 Your observation is correct, as of the ENDES 2017 the specific question was considered to know the ethnicity of the respondents. However, since it is an important variable for our study, we consider those women surveyed in 2015 and 2016 as Mestizas if they reported that the mother tongue was Spanish, while we consider as Quechua ethnicity if the mother tongue was Quechua and as other ethnicities if they reported having another mother tongue. Additionally, we highlight such limitation in the description so that it can be taken into account by readers.

 Independent variables section: “It is important to note that for the years 2015 and 2016, the ENDES survey did not specifically collect information on ethnicity. As a result, in this study, an alternative approach was taken to approximate ethnicity by considering the information on mother tongue. Specifically, individuals who reported that their mothers spoke Spanish were categorized as mestizos. Those who reported speaking the Quechua language were categorized as Quechua. Individuals who reported other languages were classified under the category of other ethnicities. It is important to acknowledge this limitation when interpreting the results related to ethnicity in the study.”

 Response #5

 We appreciate your comment.

 We add the relevant information to better clarify the process of obtaining the information.

 Population, smaple, and sampling section: “It is important to highlight that the DHS implemented a face-to-face survey methodology, specifically targeting women of reproductive age (15 to 49 years in the 2015, 2016, and 2017 DHS, and 12 to 49 years in the 2018 to 2019 DHS). Within the scope of the ENDES, the survey of women in this age group was carried out in a personalized, confidential, and respectful manner, without requiring the presence of parents. Moreover, it is worth noting that participants who chose not to answer a particular question were recorded as having missing data. This approach aimed to ensure the privacy and comfort of the respondents, fostering an environment where they could provide accurate and honest responses.”

 Response #6

 We appreciate your comment.

 We added multicollinearity assessment as part of the statistical analyses. 

 “To assess collinearity, the variance inflation factor (VIF) was used, where a value > 10 determined multicollinearities between variables; however, all values obtained were less than 10.”

 Response #7

 We appreciate your comment.

 We reconstructed Figure 1 to clarify the selection process.

 Response #8

 We appreciate your comment.

 We reconstructed Figure 1 to clarify the selection process.

 The corresponding doubt corresponded to the total number of respondents with information on the indicated item, but as it was confusing, we decided to change the flow chart.

 Response #9

 We appreciate your comment.

 With respect to the culture of ethnic groups, we found that ethnic groups have a higher level of fertility and lower use of contraceptive methods, as well as a higher acceptance of marriage.

 This information is found in the fourth paragraph of the discussion. “In Peru in 2016, it was observed that the population of native origin (Quechua, Aymara or Amazonian origin) had a higher level of fertility and a lower proportion of contraceptive methods used. 27 In these ethnic groups, a greater acceptance of early marriage and pregnancy has also been reported. 25 Therefore, greater state intervention is required in these population groups to reduce the gaps in access to sexual and reproductive health information for adolescents.”

 With respect to the type of family, the types of cohabitation, we consider that we do not have enough information based on our findings to provide more information on the subject, since we only consider the fact of being with or without a partner (married/cohabiting).
